# Psychiatric Hospitalization for Psychopathological Disorders and Self-Injurious Behaviors in Italian Children and Adolescents during COVID-19

**DOI:** 10.3390/children10121846

**Published:** 2023-11-24

**Authors:** Maria Pontillo, Deny Menghini, Roberto Averna, Milena Labonia, Giulia Lazzaro, Maria Cristina Tata, Stefano Vicari

**Affiliations:** 1 Child & Adolescent Neuropsychiatry Unit, Bambino Gesù Children’s Hospital, IRCCS, 00146 Rome, Italy; deny.menghini@opbg.net (D.M.); roberto.averna@opbg.net (R.A.); milena.labonia@opbg.net (M.L.); giulia.lazzaro@opbg.net (G.L.); mariacristina.tata@opbg.net (M.C.T.); stefano.vicari@opbg.net (S.V.); 2 Department of Life Sciences and Public Health, Università Cattolica del Sacro Cuore, 00168 Rome, Italy

**Keywords:** COVID-19 pandemic, children, adolescents, psychiatric hospitalization, mood disorders, non-suicidal self-injurious behaviors, suicidal ideation

## Abstract

The evidence shows that the COVID-19 pandemic dramatically increased the number of urgent psychiatric consultations for children and adolescents in hospital emergency departments (EDs). However, what needs to be further investigated are the characteristics of psychiatric hospitalization in children and adolescents admitted to the Child and Adolescent Neuropsychiatry Unit wards in EDs. Specifically, this retrospective study aimed to examine changes in (i) the number of inpatients and (ii) the distribution of psychopathological disorders and self-injurious behaviors in our Child and Adolescent Neuropsychiatry Unit ward during the COVID-19 lockdown in Italy (March–June 2020; October 2020–January 2021) compared with the same months of previous years. We found a significantly lower number of inpatients during the first four quarantine months than the first four reference months and a higher number of inpatients during the second four quarantine months than the second four reference months. Additionally, we found an increased frequency of mood disorders, non-suicidal self-injurious behavior, and suicidal ideation during the COVID-19 lockdown compared to the reference periods. Our findings underline the need to develop psychological healthcare services for future emergency periods in order to identify and treat psychological distress in children and adolescents early, reducing the risk of psychiatric hospitalization.

## 1. Introduction

In March 2020, the novel coronavirus (i.e., COVID-19) pandemic forced the general population to engage with unprecedented social distancing and isolation measures in an effort to contain the infection. Worldwide, governments adopted strict lockdown measures to control the spread of the virus. Therefore, everyday habits and lifestyles were dramatically altered across familial, social, and professional spheres. This had a profound impact on the functioning of all individuals, regardless of their age, sex, and socio-economic status. 

Research has shown that viral outbreaks and their associated quarantine measures tend to decrease psychosocial wellbeing. During the SARS CoV1 epidemic, for example, major anxiety and depression symptoms were found to be associated with a major risk of developing post-traumatic stress disorder and abusive conduct in young adults [1,2,3]. A number of studies [4,5,6,7] have reported a widespread presence of anxiety symptoms, mood deflection, and sleep disturbance in the general population during the COVID-19 pandemic. They have also reported significant psychological effects in children and adolescents [8,9,10], manifesting differently according to age. Specifically, in preschool children (i.e., aged 3–6 years), Jiao et al. [11] found an increase in irritability, inattention, and disruptive behaviors during the COVID-19 pandemic, associated with a high incidence of sleep disturbance, agitation, and separation anxiety due to the fear that family members could contract the virus. In children and adolescents (i.e., aged 6–18 years), Uccella et al. [12] observed an association between isolation, social distancing due to COVID-19, and anxiety, fear, and uncertainty about the future. 

The COVID-19 pandemic and its associated restrictions may have particularly affected adolescents and preadolescents, who would have already been experiencing a vulnerable transitional period characterized by the onset of puberty and significant neurobiological, social, and cognitive changes [13,14]. Specifically, adolescents may have experienced aggravated chronic and acute stress during the pandemic, in the form of worrying about family members, unexpected mourning, anxiety around the sudden closure of school and home confinement, stress caused by an increased use of the internet and social media, and worry about the economic future of their family. In this vein, Vindegaard and Benros (5) suggested that the interruption of social relations during periods of school closure represented a main stressor for adolescents during the pandemic. 

Literature narrative review from Guessoum et al. [15] on adolescent mental health during the COVID-19 pandemic showed an increase in psychopathological disorders, including depressive disorders, anxiety disorders, post-traumatic stress disorder, and grief-related symptoms. In a retrospective cohort study, Ougrin et al. [16] found that the proportion of young people presenting with self-harm increased from 50% in 2019 to 57% in 2020; moreover, the proportion of young people with emotional disorders increased from 58% to 66% over the same period. 

In a recent meta-analysis, Panchal et al. (2023) examined in detail this significant existing literature on the effects of quarantine measures on the mental health of children and adolescents.

The results highlighted that anxiety and depression are the most frequently reported symptoms by children and adolescents during quarantine periods. Specifically, the prevalence of anxiety symptoms varied between 1.8% and 49.5% in the studies, with 13.4% of the examined children reporting severe anxiety. The prevalence of depressive symptoms ranged from 2.2% to 63.8% in the studies, and 27% of the children and adolescents included in the studies reported severe depression. Additionally, irritability was frequently reported among children and adolescents during quarantine periods, with a prevalence ranging from 16.7% to 73.2% in the various studies examined. Panchal et al. [17], along with another review by Theberath et al. [18], concluded by emphasizing the need for in-depth studies on the effects of lockdown on the mental health of children and adolescents. These studies should facilitate the development of appropriate guidelines for managing emergency periods like the one that has passed. In fact, most of the studies cited here primarily focus on the prevalence of psychiatric symptoms in children and adolescents during COVID-19 and associated quarantine measures without delving into the different levels of care (e.g., urgent psychiatric consultation and psychiatric hospitalization) required for these symptoms during that time. We believe that this is necessary to fully understand this impact and prepare clinical services for future periods in which significant reductions in social, school, and leisure activities may once again be enforced.

This is particularly relevant to the context of Italy, which was the first European country to adopt strict measures involving social distancing, prohibitions, and restrictions during the COVID-19 pandemic

In February 2020, the Italian government declared a state of emergency over the COVID-19 pandemic. The first phase of the government’s COVID-19 response (9 March to 3 May 2020) included a general school closure, freedom of movement restrictions (enforceable by law), and the cancellation of all non-essential trips (e.g., school trips abroad). Consequently, all activities were paused, resulting in a significant reduction in child and adolescent extracurricular activities, including sports practices, music lessons, and theater classes. During the second phase of the COVID-19 response (4 May to 14 June 2020), containment measures were eased, resulting in the legal removal of social isolation and regional movement limitations and the resumption of commercial activities. However, schools remained closed, and only exams were attended in person, with restrictions. Finally, in the third stage of the COVID-19 response (15 June to 7 October 2020), all leisure activities were resumed with restrictions. 

The second wave of COVID-19 diffusion was observable in Italy from 8 October 2020. At this time, the Italian government once again applied strong limitations, closing schools and non-essential services. Furthermore, beginning on 6 November 2020, the Italian government, following European Community directions, applied regional containment measures, based on the regional contagious index (i.e., the Rt index). At that time, school attendance was limited to a percentage capacity (e.g., 50% of all students attending at a time). Finally, from 7 January 2021, regional restrictions continued, enforceable via colored bands for each region.

Previous retrospective studies conducted by our research group have focused on the increase in urgent psychiatric consultations for children and adolescents during the COVID-19 pandemic in Italy. In particular, Serra et al. [19] demonstrated that urgent psychiatric consultations for any reason and for suicide attempts significantly reached their peak during the second quarantine period in Italy. However, this study examines data from our emergency department and does not take into account any changes in the psychiatric hospitalization of children and adolescents during the quarantine periods. We believe that this should be investigated in light of the prolonged period of home confinement, school closures, and social restrictions experienced by Italian children and adolescents. Based on these considerations, our study is the first initial examination of the characteristics of psychiatric hospitalization in Italian children and adolescents during COVID-19. Specifically, in the current retrospective study, we examined changes in the number of admissions to the Child and Adolescent Neuropsychiatry Unit ward of Bambino Gesù Children’s Hospital and the presentation of psychopathological disorders (i.e., psychosis, mood disorder, post-traumatic stress disorder, and behavior disorder) and self-injurious behaviors in the admitted patients during the COVID-19 quarantine periods in Italy, in comparison to reference periods. Specifically, admissions during the months in which quarantine was imposed in Italy (i.e., March to June 2020; October 2020 to January 2021) were compared with admissions in the 12 months prior (i.e., March to June 2019; October 2019 to January 2020). 

## 2. Materials and Methods

### 2.1. Participants 

The present retrospective study examined admissions to the Child and Adolescent Neuropsychiatry Unit ward of Bambino Gesù Children’s Hospital during COVID-19 pandemic based on a retrospective chart review. Bambino Gesù Children’s Hospital is the largest pediatric hospital in Europe and a 607-bed tertiary care academic hospital in Italy (Rome). The hospital is widely recognized as a referral center for all pediatric specialties at the national and international levels. The Child and Adolescent Neuropsychiatry Unit emergency ward admits children and adolescents (i.e., aged 5–17.9 years) who have been referred from the emergency department at a maximum rate of eight beds per day. 

For our analysis, we included data on patients (a) aged 6–17.9 years and (b) who have been referred from the emergency department only once. Patients who presented with repeated evaluations and/or the revolving door phenomenon were not considered for the current study. The current study examined admissions through a retrospective analysis of medical records during two consecutive 4-month periods when a national quarantine was enforced in response to the COVID-19 pandemic in Italy: from March to June 2020 and October 2020 to January 2021. Admissions during the corresponding periods from 12 months prior (i.e., March to June 2019 and October 2019 to January 2020) were also taken into account, enabling comparisons regarding the number of admissions as well as the distribution of psychopathological disorders and self-injurious behaviors.

In our study, we considered patients with psychopathological disorders diagnosed according to DSM-5 criteria (i.e., psychosis, mood disorders, post-traumatic stress disorder, and behavior disorders) and self-injurious behaviors defined as “any act of self-poisoning or self-injury, irrespective of the underlying intent,” according to the UK National Institute for Health and Care Excellence [20] clinical guidelines. Thus, both non-suicidal self-injury and suicide attempts were included. Suicidal ideation was categorized, according to the DSM-5 [21], as “thoughts about self-harm, with deliberate consideration or planning of possible techniques of causing one’s own death”.

The study was approved by the Ethics Committee of the Children Hospital Bambino Gesù. All participants and their parents/legal guardians provided written informed assent and consent.

#### 2.1.1. Clinical Setting

In our Child and Adolescent Neuropsychiatry Unit ward, psychiatric hospitalization is conducted as follows: continuous care for admitted children and adolescents is provided by nurses and health and social workers, as parental presence within the ward is prohibited. Parents have scheduled visits with their children each afternoon, lasting approximately 2 h. Limiting parental presence serves the purpose of preventing intra-family aggression or crises and facilitating an assessment of the patient free from parental influence on the reported information. Nevertheless, parents play an integral role during their children’s hospitalization. Psychologists and neuropsychiatrists engage with parents for diagnostic evaluations and updates on their children’s clinical conditions. Psychoeducational interventions are designed to alleviate parental stress related to the diagnosis and hospitalization of their children and adolescents. Moreover, neuropsychiatrists and psychologists engage with children and adolescents admitted to daily group therapy sessions, psychological assessment tests, and individual psychological interventions. Regular team meetings are scheduled between nurses, neuropsychiatrists, and psychologists to coordinate tasks and address critical issues. Surveillance cameras are installed in all rooms to monitor any instances of self-harm or suicidal attempts; a central monitor is positioned in the nursing room. The ward also includes a common area where children participate in various activities together, such as shared meals, educational sessions conducted by teachers according to their educational level, drama classes, music listening, and movie screenings.

The primary objective of hospitalization is to address the acute phase of the disorder and to facilitate the transition to mental health departments within the local community. Certain inpatients may be transferred to specialized psychiatric facilities catering to children and adolescents experiencing severe emotional disorders and/or behavioral issues that cannot be adequately managed in outpatient settings.

#### 2.1.2. Clinical Assessment

All the patients included in this study were assessed by expert neuropsychiatrists and developmental psychologists according to DSM-5 criteria [21], based on developmental history and an extensive clinical examination. All the patients considered were evaluated via the same psychopathological assessment protocol:

- Psychopathological disorders were assessed using the Schedule for Affective Disorders and Schizophrenia for School-Aged Children [22], a semi-structured interview administered to obtain a psychopathological diagnosis according to the DSM-5 criteria. The K-SADS-PL DSM-5, as proposed in the instrument manual by Kaufman et al. [22], provides a source of information for the child/adolescent as well as the parents.
- Suicidal ideation and behavior were assessed with the Columbia–Suicide Severity Rating Scale (C-SSRS). Suicidal ideation is defined by a score of 3 or above.
- Non-suicidal self-injury (NSSI) was evaluated with an assessment of the DSM-5 categorical diagnostic criteria for non-suicidal self-injury (NSSI), that is, NSSI on at least 5 days within the past year, suicidal ideation absent or a low score (a score below 3 at the C-SSRS), and no previous suicide attempts. All these diagnostic tools were administered by trained child neuropsychiatrists and psychologists.

### 2.2. Statistical Analyses

The number of admissions was first considered for the statistical analyses and refers to the number of patients who have only accessed the Child and Adolescent Neuropsychiatry Unit ward of the Bambino Gesù Children’s Hospital once. Patients presenting revolving-door and repeated evaluations during the analyzed period were excluded. Chi-square (χ^2^) contingency tables were used to explore potential differences in the number of admissions between the first quarantine period (March to June 2020) and the first reference period (March to June 2019), as well as for the second quarantine period (October 2020 to January 2021) compared to the second reference period (October 2019 to January 2020). To verify which cells contributed to potential significant differences, calculations of residuals proposed by Sharpe et al. [23] were conducted. 

Moreover, the number of psychopathological disorders was considered for the statistical analyses, and the number of patients with psychopathological disorders as a primary diagnosis, i.e., psychosis, mood disorders, post-traumatic disorders, and behavior disorders, was calculated among the total of admissions. χ^2^ contingency tables were used, and odds ratios (ORs) with 95% confidence intervals (95% CI) were calculated to measure the associations between diagnoses (i.e., psychosis, mood disorder, post-traumatic stress disorder, and behavior disorder) and the quarantine period and the reference periods. 

The same analyses were run to explore the associations between self-injurious behaviors (i.e., suicidal ideation, non-suicidal self-injurious behaviors, and suicide attempts) and the quarantine versus the reference periods. 

A *p*-value of less than 0.05 was considered statistically significant. χ^2^ analyses and OR were computed using R Studio (R Studio, Boston, MA, USA), with particular reliance on the Epitools package. 

## 3. Results

### 3.1. Number of Admissions

The total number of admissions among these periods was 377, with an average of 6 days of hospitalization and ages ranging from 6 to 18 years old. The number of admissions to the Child and Adolescent Neuropsychiatry Unit ward differed between the quarantine and the reference periods (χ21 = 5.43, *p* = 0.0197). Specifically, z-tests were run to compare cells [23], with the critical value of 2.33 (√χ21 = √5.43). There were significantly fewer admissions during the first quarantine period than the first reference period (77 vs. 91; z-test = −2.34) and significantly more admissions during the second quarantine period than the second reference period (121 vs. 88; z-test = 2.34). 

### 3.2. Distribution of Psychopathological Disorders

For a more comprehensive overview of the psychopathological distribution trend, we analyzed the distributions of psychopathological disorders, merging those from the first quarantine period (March to June 2020) and the second quarantine period (October 2020 to January 2021), as well as the first reference period (March to June 2019) and the second reference period (October 2019 to January 2020). See Table 1. 

Overall, the results showed a significant association between mood disorders and the quarantine versus the reference periods, with significantly more diagnosed mood disorders among admitted patients during the quarantine periods (OR = 1.54, 95% CI [1.01, 2.37], *p* = 0.04). No further associations emerged between diagnoses and the quarantine period and the reference periods (psychosis: OR = 0.68, 95% CI [0.41, 1.12], *p* = 0.13; post-traumatic stress disorder: OR = 1.01, 95% CI [0.50, 2.04], *p* = 0.97; behavioral disorders: OR = 0.50, 95% CI [0.19, 1.21], *p* = 0.12). 

The distributions of psychopathological disorders (i.e., psychosis, mood disorder, post-traumatic stress disorder, and behavior disorder) between the first quarantine period (March to June 2020) and the first reference period (March to June 2019), as well as for the second quarantine period (October 2020 to January 2021) and the second reference period (October 2019 to January 2020), are presented in the Supplementary Materials (Table S1). 

### 3.3. Distribution of Self-Injurious Behaviors

For a more comprehensive overview of the self-injurious behaviors trend, we analyzed the distributions of self-injurious behaviors (i.e., suicidal ideation, non-suicidal self-injurious behaviors, and suicide attempts), merging those from the first quarantine period (March to June 2020) and the second quarantine period (October 2020 to January 2021), as well as the first reference period (March to June 2019) and the second reference period (October 2019 to January 2020). See Table 2. 

The results showed a significant association between suicidal ideation and the quarantine and the reference periods, with significantly more suicidal ideation among admitted patients during the quarantine periods (OR = 1.61, 95% CI [1.05, 2.48], *p* = 0.03). Furthermore, a significant association emerged between non-suicidal self-injurious behaviors and the quarantine and the reference periods, revealing a greater frequency of non-suicidal self-injurious behaviors among admitted patients during the quarantine periods (OR = 1.87, 95% CI [1.24, 2.82], *p* = 0.003). No significant association was found with reference to suicide attempts (OR = 0.88, 95% CI [0.52, 1.5], *p* = 0.65). 

The distributions of self-injurious behaviors (i.e., suicidal ideation, non-suicidal self-injurious behaviors, and suicide attempts) between the first quarantine period (March to June 2020) and the first reference period (March to June 2019), as well as for the second quarantine period (October 2020 to January 2021) and the second reference period (October 2019 to January 2020), are presented in Supplementary Materials (Table S2).

## 4. Discussion

The present retrospective study examined changes in the number of direct admissions to the Child and Adolescent Neuropsychiatry Unit emergency ward of Bambino Gesù Children’s Hospital and the distribution of psychiatric disorders and self-injurious behaviors among admitted patients during the COVID-19 quarantine in Italy, in comparison to a reference period. 

The results showed a decrease in the number of direct admissions during the first quarantine period. 

Specifically, the number of admissions during the first quarantine period (77 patients, from March to June 2020) was lower than that of the first reference period (91 patients, from March to June 2019). This finding aligns with the results of a retrospective international cohort study [16] of children and adolescent inpatients in hospital emergency departments in European countries (i.e., England, Scotland, Ireland, Hungary, Turkey, etc.). 

There are two potential explanations for the lower number of admissions during the first quarantine period. The first explanation is that families may have avoided emergency departments for fear of becoming infected. Our hypothesis is consistent with the results of previous studies [24,25], which have underlined that the worries of young people and their parents about contracting and spreading COVID-19 may have severely reduced young people’s psychological wellbeing and the use of mental health services. The second explanation, as noted by Ougrin et al. [16], is that the lower number of children and adolescent admissions to psychiatry wards may be explained by the quarantine measures at the time. In Italy, many children and adolescents stopped attending school in March 2020. This is likely to have reduced their experience of academic pressure and minimized their number of distress factors. For example, many children and adolescents may have had fewer or no face-to-face relationships, which the literature [23] associates with an increased risk of self-injurious behaviors and psychopathological disorders, as well as bullying and peer pressure to abuse alcohol and drugs. In addition, many children and adolescents may have increased their psychological wellbeing during the first quarantine period as a result of spending more time with family. 

Accordingly, it may be that some children and adolescents who would have otherwise accessed the hospital in crisis were able to access alternative coping strategies linked to different habits (i.e., staying at home) during the lockdown. However, staying at home may have also generated long-term negative effects on the mental health of children and adolescents. In line with this, the present study found that the number of direct admissions increased significantly during the second quarantine period compared to the second reference period (i.e., 121 patients from October 2020 to January 2021 vs. 88 patients from October 2019 to January 2020). It is possible that, in the second quarantine period, the persistent isolation, social distancing, and homeschooling may have determined anxiety, distress, fear, and a low mood, while reducing the ability to perform coping strategies and increasing the need for psychiatric services. In addition, differently from the first quarantine period, in the second quarantine period, parents went back to work, and they were thus less able to provide physical and emotional support to their children, who nevertheless continued to stay at home. 

Considering the distribution of psychopathological disorders among the child and adolescent admissions during the quarantine periods, mood disorders were the most common. Additionally, these were more frequent during the quarantine periods than the reference periods (69.2% vs. 59.2%, respectively). These results are compatible with the literature [25,26,27,28], which indicates that the persistence of social restrictions and isolation during the COVID-19 pandemic was a risk factor for psychopathological disorders (including mood disorders) among children and adolescents. Oliva et al. [29], in a prospective cross-sectional study involving 9688 pre-adolescents and adolescents, showed that the lifestyle changes generated by enforced and prolonged social isolation were associated with an increased prevalence of mental disorders, including mood disorders. In particular, the reduction in physical activity, the increased screen exposure (including for the purposes of homeschooling), and the interruption of face-to-face relationships with peers (which fostered a dependence on social media that contributed to the excessive screen exposure) were identified as risk factors for the psychological wellbeing of children and adolescents during COVID-19. Additionally, changes in sleeping patterns (e.g., irregular wake-up times and shorter or longer periods of sleep) were also acknowledged as a risk factor [30]. 

Concerning self-injurious behaviors among the admitted patients during the quarantine periods, an increased frequency of non-suicidal self-injurious behaviors was observed compared to the reference periods (i.e., 58.6% during the quarantine periods vs. 43.2% during the reference periods). This finding aligns with the results of previous studies of adolescents during COVID-19 [16,31,32,33]. As proposed by Plener [30], non-suicidal self-injurious behaviors are often applied as an emotion regulation strategy to temporarily decrease or eliminate negative emotions. Therefore, negative emotions activated by lockdown pressure may have represented a significant risk factor for non-suicidal self-injurious behaviors during the quarantine. Additionally, during the quarantine periods, social support, which has been identified as a strong protective factor against non-suicidal self-injurious behaviors [34,35], may have only been accessible to some children and adolescents through social media. These factors, alongside limited access to face-to-face professional support, may have amplified children and adolescents’ need to regulate negative emotions, and thus increased their likelihood of practicing non-suicidal self-injurious behaviors. In line with the literature [36,37,38], the present findings could be helpful in informing the development of clinical and educational intervention programs for non-suicidal self-injurious behaviors during quarantine periods.

Moreover, Asarnow et al. [39] described non-suicidal self-injurious behaviors as a significant predictor of suicidal ideation and suicide attempts in adolescence. Consistent with this, the present study found increased suicidal ideation amongst children and adolescent admissions during the quarantine periods (i.e., 40.4% during the quarantine periods vs. 29.6% during the reference periods). However, no increase in suicidal attempts during the quarantine periods was observed (i.e., 17.2% during the quarantine periods vs. 18.9% during the reference periods). As a potential explanation for this, home confinement may have facilitated child–parent communication and intensified parental surveillance, thereby minimizing suicide attempts.

At the time of writing, the present study represented the first to assess the characteristics of psychiatric hospitalization, such as the number of inpatients admitted, psychopathological disorders, and self-injurious behavior, in children and adolescents during the two COVID-19 quarantine periods in Italy. Regarding the limitations of our study, this research did not delve into the relationship between psychopathological disorders or self-injurious behaviors detected during hospitalization and any risk factors. These risk factors could be clinical (e.g., psychiatric family history or prior psychiatric diagnoses), individual (e.g., temperament traits or personality characteristics), or environmental (emotional or physical abuse within the family and intra-family conflicts). These factors, along with lifestyle changes associated with quarantine measures (e.g., changes in sleep patterns due to home confinement, homeschooling, and decreased face-to-face social interaction), should be considered in future research. Furthermore, future studies could investigate the long-term outcomes for children and adolescents hospitalized during the COVID-19 pandemic. It would be interesting to examine whether there is a chronicity of the mood disorders identified and the clinical progression of the self-injurious behaviors. This would allow for further reflections on interventions, both preventive and therapeutic, to be implemented in emergency situations.

## 5. Conclusions

The present findings offer valuable insights into the impact of the COVID-19 pandemic and quarantine measures on psychiatric hospitalization among Italian children and adolescents. Our results highlight changes in admission patterns during the COVID-19 pandemic and suggest a critical examination of the psychological needs of young individuals during emergency situations. Children and adolescents are particularly susceptible to the psychological effects of the COVID-19 pandemic due to their developing ability to regulate emotions and cope with stressful life events. Consequently, they may exhibit negative psychological outcomes requiring psychiatric hospitalization. In our sample of Italian children and adolescents, we found an increased frequency of mood disorders, non-suicidal self-injurious behavior, and suicidal ideation during the COVID-19 lockdown compared to reference periods. Although with caution, we hypothesize that the rapid spread of COVID-19 in Italy with numerous victims and the consequent prolonged period of home isolation with the destruction of daily routines, the closure of schools, and the absence of in-person relationships have represented factors precipitating the onset of mood disorders in probably already vulnerable young people. Moreover, despite the implementation of home-schooling with the possibility of meeting teachers online and the presence of parents at home, it should be considered that the negative emotional atmosphere and fear of death present both in the school and family contexts have hindered the long-term adaptation of young people to the pandemic and the quarantine measures necessary for its containment.

In times of emergency, supportive therapeutic programs can prevent the distress of hospitalization and separation from home and family. Drawing from the cooperative model proposed by Brofenbrenner [40] and Zhou [41], we propose psychological interventions involving three cooperative systems: the social system, the school system, and the family system. The social system (government and social organizations) could implement psychological screening programs (via social media, online chat, or telephone) to identify and address early signs of anxiety, depression, and emotional dysregulation among children and adolescents experiencing a pandemic and associated quarantine measures.

Simultaneously, involving parents and teachers in these psychological support programs is essential to alleviate their emotional distress, enhance their coping strategies, and help children and adolescents. These support programs should be accessible during both the acute and post-pandemic stages. In the acute phase, they can shield children and adolescents from psychological and behavioral crises that might lead to psychiatric hospitalization. In the post-pandemic phase, these programs serve to protect them from enduring long-term psychological difficulties that could potentially progress to diagnosable psychiatric disorders.

## Figures and Tables

**Table 1:** Distribution of psychopathological diagnoses between the quarantine and the reference periods.

	Distribution among Admitted Patients	
Diagnosis	Quarantine Periods	Reference Periods
	# (%)	# (%)
Psychosis	34 (17.2)	42 (23.5)
Mood disorders	137 (69.2)	106 (59.2)
Post-traumatic stress disorder	19 (9.6)	17 (9.5)
Behavior disorders	8 (4.0)	14 (7.8)

**Table 2:** Distribution of self-injurious behaviors between the quarantine and the reference periods.

	Distribution among Admitted Patients	
Self-Injurious Behaviors	Quarantine Periods	Reference Periods
	# (%)	# (%)
Suicidal ideation	80 (40.4)	53 (29.6)
Non-suicidal self-injurious behaviors	116 (58.6)	77 (43.2)
Suicide attempts	34 (17.2)	34 (18.9)

## Data Availability

The raw data that underpins the conclusions of this article will be provided by the corresponding author upon reasonable request. The data is not publicly available due to the nature of the information and the regulations governing the retrospective study mode, which involves the review of medical records.

## Supplementary Materials

The following supporting information can be downloaded at https://www.mdpi.com/article/10.3390/children10121846/s1. Table S1. Distribution of psychopathological diagnoses between the first quarantine period, first reference period, second quarantine period, and the second reference period. Table S2. Distribution of self-injurious behaviors between the first quarantine period, first reference period, second quarantine period, and the second reference period.

## References

[1] Hawryluck L., Gold W.L., Robinson S., Pogorski S., Galea S., Styra R. (2004). SARS Control and Psychological Effects of Quarantine, Toronto, Canada. Emerg. Infect. Dis..

[2] Reynolds D.L., Garay J.R., Deamond S.L., Moran M.K., Gold W., Styra R. (2008). Understanding, compliance and psychological impact of the SARS quarantine experience. Epidemiol. Infect..

[3] Brooks S.K., Smith L.E., Webster R.K., Weston D., Woodland L., Hall I., Rubin G.J. (2020). The impact of unplanned school closure on children’s social contact: Rapid evidence review. Eurosurveillance. Euro Surveill..

[4] Torales J., O’Higgins M., Castaldelli-Maia J.M., Ventriglio A. (2020). The outbreak of COVID-19 coronavirus and its impact on global mental health. Int. J. Soc. Psychiatry.

[5] Vindegaard N., Benros M.E. (2020). COVID-19 pandemic and mental health consequences: Systematic review of the current evidence. Brain Behav. Immun..

[6] Racine N., McArthur B.A., Cooke J.E., Eirich R., Zhu J., Madigan S. (2021). Global Prevalence of Depressive and Anxiety Symptoms in Children and Adolescents During COVID-19: A Meta-analysis. JAMA Pediatr..

[7] Wolf K., Schmitz J. (2023). Scoping review: Longitudinal effects of the COVID-19 pandemic on child and adolescent mental health. Eur. Child. Adolesc. Psychiatry.

[8] Shen K., Yang Y., Wang T., Zhao D., Jiang Y., Jin R., Zheng Y., Xu B., Xie Z., Lin L. (2020). Diagnosis, treatment, and prevention of 2019 novel coronavirus infection in children: Experts’ consensus statement. World J. Pediatr..

[9] Meherali S., Punjani N., Louie-Poon S., Abdul Rahim K., Das J.K., Salam R.A., Lassi Z.S. (2021). Mental Health of Children and Adolescents Amidst COVID-19 and Past Pandemics: A Rapid Systematic Review. Int. J. Environ. Res. Public Health.

[10] Samji H., Wu J., Ladak A., Vossen C., Stewart E., Dove N., Long D., Snell G. (2022). Review: Mental health impacts of the COVID-19 pandemic on children and youth—A systematic review. Child. Adoles Ment. Health.

[11] Jiao W.Y., Wang L.N., Liu J., Fang S.F., Jiao F.Y., Pettoello-Mantovani M., Somekh E. (2020). Behavioral and Emotional Disorders in Children during the COVID-19 Epidemic. J. Pediatr..

[12] Uccella S., De Grandis E., De Carli F., D’Apruzzo M., Siri L., Preiti D., Di Profio S., Rebora S., Cimellaro P., Biolcati Rinaldi A. (2021). Impact of the COVID-19 Outbreak on the Behavior of Families in Italy: A Focus on Children and Adolescents. Front. Public Health.

[13] Larsen B., Luna B. (2018). Adolescence as a neurobiological critical period for the development of higher-order cognition. Neurosci. Biobehav. Rev..

[14] Best O., Ban S. (2021). Adolescence: Physical changes and neurological development. Br. J. Nurs..

[15] Guessoum S.B., Lachal J., Radjack R., Carretier E., Minassian S., Benoit L., Moro M.R. (2020). Adolescent psychiatric disorders during the COVID-19 pandemic and lockdown. Psychiatr. Res..

[16] Ougrin D., Wong B.H., Vaezinejad M., Plener P.L., Mehdi T., Romaniuk L., Barrett E., Hussain H., Lloyd A., Tolmac J. (2022). Pandemic-related emergency psychiatric presentations for self-harm of children and adolescents in 10 countries (PREP-kids): A retrospective international cohort study. Eur. Child. Adolesc. Psychiatr..

[17] Panchal U., Salazar de Pablo G., Franco M., Moreno C., Parellada M., Arango C., Fusar-Poli P. (2023). The impact of COVID-19 lockdown on child and adolescent mental health: Systematic review. Eur. Child. Adolesc. Psychiatry.

[18] Theberath M., Bauer D., Chen W., Salinas M., Mohabbat A.B., Yang J., Chon T.Y., Bauer B.A., Wahner-Roedler D.L. (2022). Effects of COVID-19 pandemic on mental health of children and adolescents: A systematic review of survey studies. SAGE Open Med..

[19] Serra G., Apicella M., Iannoni M.E., Trasolini M., Andracchio E., Chieppa F., Averna R., Guidetti C., Maglio G., Reale A. (2023). Urgent Psychiatric Consultations for Suicidal Ideation and Behaviors in Italian Adolescents during Different COVID-19 Pandemic Phases. J. Pers. Med..

[20] National Collaborating Centre for Mental Health (UK) (2004). Self-Harm: The Short-Term Physical and Psychological Management and Secondary Prevention of Self-Harm in Primary and Secondary Care.

[21] American Psychiatric Association (2013). Diagnostic and Statistical Manual of Mental Disorders: DSM-5.

[22] Kaufman J., Birmaher B., Rao U., Ryan N. (2019). K-SADS-PL DSM-5^®^. Intervista Diagnostica Per la Valutazione dei Disturbi Psicopatologici in Bambini e Adolescenti.

[23] Sharpe D. (2023). Chi-Square Test is Statistically Significant: Now What?. Pract. Assess Res. Evaluation.

[24] Crawley E., Loades M., Feder G., Logan S., Redwood S., Macleod J. (2020). Wider collateral damage to children in the UK because of the social distancing measures designed to reduce the impact of COVID-19 in adults. BMJPO.

[25] Munro A.P.S., Faust S.N. (2020). Children are not COVID-19 super spreaders: Time to go back to school. Arch Dis. Child..

[26] Lazuras L., Barkoukis V., Tsorbatzoudis H. (2017). Face-to-face bullying and cyberbullying in adolescents: Trans-contextual effects and role overlap. Technol. Soc..

[27] Viner R.M., Russell S.J., Croker H., Packer J., Ward J., Stansfield C., Mytton O., Bonell C., Booy R. (2020). School closure and management practices during coronavirus outbreaks including COVID-19: A rapid systematic review. Lancet Child. Adolesc. Health.

[28] Montreuil M., Camden C., Genest C., Gilbert E., Laberge-Perrault E., Piché G., Rassy J., Bogossian A., Gendron-Cloutier L., Barbo G. (2023). Children and adolescents’ mental health in pandemics and confinement: A scoping review of vulnerability factors and repercussions. J. Child Health Care.

[29] Oliva S., Russo G., Gili R., Russo L., Di Mauro A., Spagnoli A., Alunni Fegatelli D., Romani M., Costa A., Veraldi S. (2021). Risks and Protective Factors Associated With Mental Health Symptoms During COVID-19 Home Confinement in Italian Children and Adolescents: The #Understandingkids Study. Front. Pediatr..

[30] Amran M.S. (2022). Psychosocial risk factors associated with mental health of adolescents amidst the COVID-19 pandemic outbreak. Int. J. Soc. Psychiatry.

[31] Tang W.C., Lin M.P., You J., Wu J.Y.W., Chen K.C. (2023). Prevalence and psychosocial risk factors of nonsuicidal self-injury among adolescents during the COVID-19 outbreak. Curr. Psychol..

[32] Mucci M., Lenzi F., D’Acunto G.M., Gazzillo M., Accorinti I., Boldrini S., Distefano G., Falcone F., Fossati B., Giurdanella Annina R. (2022). How COVID-19 Phases Have Impacted Psychiatric Risk: A Retrospective Study in an Emergency Care Unit for Adolescents. Children.

[33] Plener P.L. (2021). COVID-19 and Nonsuicidal Self-Injury: The Pandemic’s Influence on an Adolescent Epidemic. Am. J. Public Health.

[34] Christoffersen M.N., Møhl B., DePanfilis D., Vammen K.S. (2015). Non-Suicidal Self-Injury—Does social support make a difference? An epidemiological investigation of a Danish national sample. Child. Abuse Negl..

[35] Xin M., Luo S., She R., Yu Y., Li L., Wang S., Ma L., Tao F., Zhang J., Zhao J. (2020). Negative cognitive and psychological correlates of mandatory quarantine during the initial COVID-19 outbreak in China. Am. Psychol..

[36] Clarke S., Allerhand L.A., Berk M.S. (2019). Recent advances in understanding and managing self-harm in adolescents. F1000Research.

[37] Olfson M., Wall M., Wang S., Crystal S., Bridge J.A., Liu S.M., Blanco C. (2018). Suicide After Deliberate Self-Harm in Adolescents and Young Adults. Pediatrics.

[38] Wilkinson P., Kelvin R., Roberts C., Dubicka B., Goodyer I. (2011). Clinical and Psychosocial Predictors of Suicide Attempts and Nonsuicidal Self-Injury in the Adolescent Depression Antidepressants and Psychotherapy Trial (ADAPT). Am. J. Psychiatry.

[39] Asarnow J.R., Porta G., Spirito A., Emslie G., Clarke G., Wagner K.D., Vitiello B., Keller M., Birmaher B., McCracken J. (2011). Suicide Attempts and Nonsuicidal Self-Injury in the Treatment of Resistant Depression in Adolescents: Findings from the TORDIA Study. J. Am. Acad. Child. Adolesc. Psychiatry.

[40] Bronfenbrenner U. (2005). The bioecological theory of human development. Making Humans Being Human.

[41] Zhou X. (2020). Managing psychological distress in children and adolescents following the COVID-19 epidemic: A cooperative approach. Psychol. Trauma.

