# Supplementary material for: Psychiatric Hospitalization for Psychopathological Disorders and Self-Injurious Behaviors in Italian Children and Adolescents during COVID-19
Source: Children (Basel). 2023 Nov 24;10(12):1846. doi: 10.3390/children10121846 (PMC10741422; doi:10.3390/children10121846)
Supplement: Supplementary file 1 [file children-10-01846-s001.zip › children-2684958-supplementary.pdf]

Table S1. Distribution of psychopathological diagnoses between the first quarantine period, first reference period, second quarantine period, and the second reference period.

Diagnosis	**Distribution among Admitted Patients**			
	**First Quarantine Period (March to June 2020)**	**First Reference Period (March to June 2019)**	**Second Quarantine Period (October 2020 to January 2021)**	**Second Reference Period (October 2019 to January 2020)**
	**# (%)**	**# (%)**	**# (%)**	**# (%)**
Psychosis	14 (16.7)	21 (19.4)	20 (16.0)	21 (20.6)
Mood disorders	49 (58.3)	48 (44.4)	85 (68.0)	50 (49.0)
Post-traumatic stress disorder	10 (11.9)	16 (14.8)	13 (10.4)	12 (11.8)
Behavior disorders	23 (13.1)	11 (21.3)	7 (5.6)	19 (18.6)

Table S2. Distribution of self-injurious behaviors between the first quarantine period, first reference period, second quarantine period, and the second reference period.

**Self-Injurious Behaviors**	**Distribution among Admitted Patients**			
	**First Quarantine Period (March to June 2020)**	**First Reference Period (March to June 2019)**	**Second Quarantine Period (October 2020 to January 2021)**	**Second Reference Period (October 2019 to January 2020)**
	**# (%)**	**# (%)**	**# (%)**	**# (%)**
Non-suicidal self-injurious behaviors	45 (56.3)	37 (46.3)	71 (47.3)	40 (47.6)
Suicidal ideation	24 (30)	21 (26.3)	56 (37.3)	32 (38.1)
Suicide attempts	11 (13.8)	22 (27.5)	23 (15.3)	12 (14.3)
